# Lung ultrasound versus chest X-ray in pediatric lower respiratory tract infections at a tertiary center: a prospective observational study

**DOI:** 10.1186/s13052-026-02314-6

**Published:** 2026-07-23

**Authors:** Rehab Elmeazawy, Asmaa Azmy Aborady, Ahmed Mohamed Abd-Razik, Adel Ali Erfan, Mohamed Adel Eltomey, Mohamed Bassiony Hamza

**Affiliations:** 1 Department of Pediatrics, Faculty of Medicine, Tanta University, Tanta, Egypt; 2 Department of Radiodiagnosis, Faculty of Medicine, Tanta University, Tanta, Egypt

**Keywords:** Lung ultrasound, Chest X-ray, Pediatric, Lower respiratory tract infection

## Abstract

**Background:**

Lower respiratory tract infections (LRTIs) are a major cause of morbidity in children. While chest X-ray (CXR) is widely used for diagnosis, it has limitations including radiation exposure and suboptimal sensitivity. Lung ultrasound (LUS) is an emerging rapid, non-invasive alternative. This study aimed to evaluate the diagnostic performance of LUS versus CXR in pediatric LRTIs, with a focus on subgroup variability.

**Methods:**

This prospective observational study included 338 children (aged 1 month–18 years) with suspected LRTIs admitted to a tertiary care hospital. All patients underwent clinical assessment, CXR, and LUS at admission and follow-up. LUS findings were compared with CXR using sensitivity, specificity, predictive values, accuracy, and Cohen’s kappa (κ) for agreement.

**Results:**

LUS demonstrated high sensitivity for pulmonary pathology at admission (95.6%) and follow-up (96.7%), with specificity improving from 29.4% at admission to 62.9% at follow-up. Overall agreement between LUS and CXR progressed from fair at admission (Cohen’s κ = 0.311, 95% CI: 0.198–0.424) to substantial at follow-up (Cohen’s κ = 0.607, 95% CI: 0.501–0.713). Subgroup analysis demonstrated meaningful variation in LUS–CXR agreement across diagnostic categories. The highest concordance was observed in pneumonia (κ = 0.438–0.470, moderate agreement), consistent with LUS’s established strength in detecting parenchymal consolidation. Agreement was notably lower in recurrent wheezing (κ = 0.208–0.283, fair agreement) and was poor to negligible in acute bronchiolitis (κ = −0.029–0.000) and the heterogeneous “others” group (κ = −0.053–0.182). LUS demonstrated high accuracy in detecting consolidations, subpleural lesions, and pleural abnormalities, and facilitated serial monitoring of disease progression during hospitalization.

**Conclusion:**

LUS is a promising, radiation-free imaging modality for the diagnosis and follow-up of pediatric LRTIs, particularly pneumonia, demonstrating moderate to substantial agreement with CXR. Its lower specificity and reduced performance in airway-predominant conditions highlight the importance of cautious interpretation alongside clinical findings.

**Supplementary Information:**

The online version contains supplementary material available at 10.1186/s13052-026-02314-6.

## Background

Lower respiratory tract infections (LRTIs) are a leading cause of morbidity and mortality in children under five, contributing to high hospitalization rates worldwide [1]. Imaging is crucial for detecting and evaluating pediatric respiratory diseases, but interpretation is challenging due to age-related anatomical differences and diverse disease presentations [2].

Plain Chest Radiography (CXR) has traditionally served as the primary imaging modality for evaluating suspected lower respiratory tract diseases. Despite its central role, CXR is hindered by significant limitations, such as high interobserver variability and a lack of sensitivity when detecting early-stage or minor pulmonary lesions. Furthermore, the risks associated with ionizing radiation exposure can lead to broader systemic issues, including increased healthcare expenditures, delayed clinical evaluations, and the potential for inappropriate antibiotic prescriptions [3].

Chest computed tomography (CT) is considered the gold standard for detailed imaging of pulmonary pathology, as it can detect small consolidations and subtle parenchymal abnormalities that may not be visible on CXR. However, routine use of CT in children is restricted due to high radiation exposure, cost, and the logistical challenges associated with transporting critically ill patients [4].

Consequently, there is a prioritized effort in pediatric medicine to adopt non-ionizing diagnostic alternatives that align with modern radiation safety standards. LUS has emerged as a superior alternative in this regard. LUS is a rapid, radiation-free, and repeatable bedside tool that can be effectively utilized by trained clinicians, offering a safer and more dynamic approach to pediatric respiratory imaging [5, 6].

Therefore, the aim of this study was to evaluate the role of lung ultrasound as a diagnostic and follow-up tool in pediatric LRTIs, with particular emphasis on comparing its performance against CXR across predefined clinical subgroups, including pneumonia, acute bronchiolitis, recurrent wheezing, and complex or rare pulmonary conditions.

## Methods

### Study design and setting

This prospective observational study was conducted on children with lower respiratory tract infections who were admitted to the Pediatric Pulmonology Unit at Tertiary Care Hospital between September 2022 and September 2024. This study was performed in line with the principles of the Declaration of Helsinki. Approval was granted by the research ethics committee of the Faculty of Medicine under the number 35,612/7/22. Written informed consent was obtained from the patients’ guardians.

### Study population

Children aged 1 month to 18 years presenting with clinical features suggestive of LRTI were consecutively enrolled, yielding a total of 338 patients categorized into four groups: pneumonia (*n* = 209), acute bronchiolitis (*n* = 45), recurrent wheezing (*n* = 48), and an “others” group (*n* = 36). The “others” group was included to capture a spectrum of less common or complex pulmonary conditions that may mimic or coexist with typical LRTI.

Inclusion criteria encompassed children within the specified age range who exhibited signs and symptoms of LRTI [7], such as cough, fever, tachypnea, respiratory distress, or abnormal chest auscultation, and for whom both lung ultrasound and CXR were available at admission. Children with significant comorbidities (cardiac, endocrine, or genetic disorders), incomplete clinical or imaging data, or absence of both LUS and CXR at admission were excluded. Patients with hemodynamic instability or lack of consent were also excluded.

### Clinical assessment

All patients underwent detailed history taking and physical examination on admission, including assessment of age, sex, presenting symptoms, and vital signs. The final clinical diagnosis was established by the treating pediatric team based on clinical presentation, laboratory results, and imaging findings.

### Chest X-ray examination

CXR was performed for all enrolled patients on admission and during follow-up using standard posteroanterior or anteroposterior views according to patient age and clinical condition. Radiographs were interpreted by experienced radiologists who were blinded both to lung ultrasound findings and to the detailed clinical and laboratory data, to minimize bias in image interpretation. Abnormal findings included consolidation, interstitial infiltrates, hyperinflation, atelectasis, and pleural effusion. CXR was used as the pragmatic reference standard for comparison with LUS, recognizing that neither CXR nor LUS perfectly captures all pulmonary pathology.

### Lung ultrasound examination

Lung ultrasound was performed on admission and during follow-up using a portable ultrasound machine equipped with a high-frequency linear probe (7–12 MHz) and, when needed, a low-frequency convex probe (3–5 MHz), with all examinations conducted at the bedside by two trained pediatric pulmonologists and one radiologist, all blinded to the chest X-ray findings. Each hemithorax was systematically scanned across 6 distinct zones (anterior upper/lower, lateral upper/lower, and posterior upper/lower) according to a standardized pediatric lung ultrasound protocol. Each zone was swept both longitudinally and transversely for a minimum of one complete respiratory cycle. All findings were documented on a structured reporting sheet per zone and classified into predefined sonographic patterns: A-lines (normal aeration); B-lines (≥ 3 per intercostal space, indicating interstitial syndrome); subpleural consolidations (< 1 cm); frank consolidations (≥ 1 cm, with or without air bronchograms); pleural effusion; tissue-like (hepatization) pattern; decreased or absent lung sliding; and advanced signs including the barcode/stratosphere sign, heterogeneous hypoechoic areas, and hypoechoic lesions [8, 9]. Bilateral findings were subsequently integrated to yield a final LUS diagnostic classification. The full scanning protocol is illustrated in Supplementary Figure S1.

All patients underwent clinical assessment, chest radiography, and lung ultrasound at admission. Follow-up LUS and CXR examinations were performed prior to hospital discharge as part of the clinical reassessment of treatment response and disease resolution.

Interobserver variability between the trained operators performing lung ultrasound was assessed using Cohen’s kappa (κ) statistics. To determine the level of diagnostic agreement between LUS and CXR, Cohen’s Kappa coefficients were calculated for the total study population and for specific diagnostic subgroups at both admission and follow-up.

### Statistical analysis

Data were analyzed using SPSS version 23 (IBM Corp., Armonk, NY, USA). Continuous variables are presented as mean ± standard deviation (SD) or median with interquartile range (IQR), and categorical variables as frequencies and percentages. Comparisons between diagnostic groups were performed using one-way ANOVA or Kruskal-Wallis tests for continuous variables and Chi-square or Fisher’s exact tests for categorical variables, as appropriate. The diagnostic performance of LUS versus CXR was evaluated using sensitivity, specificity, positive and negative predictive values (PPV, NPV), overall accuracy, and Cohen’s kappa (κ) to assess interobserver agreement and agreement between modalities. The strength of agreement was interpreted using the Landis and Koch scale, where κ value of 0.21–0.40 indicates fair agreement, 0.41–0.60 indicates moderate agreement, and 0.61–0.80 indicates substantial agreement. Paired LUS–CXR comparisons were analyzed using McNemar’s test to assess directional asymmetry in classification, with Fisher’s exact test applied when expected cell counts were < 5. Sensitivity, specificity, PPV, and NPV were calculated with Wilson score 95% confidence intervals, while overall accuracy was estimated using Clopper–Pearson exact 95% confidence intervals. Cohen’s kappa was reported with Fleiss asymptotic 95% confidence intervals for the full cohort and bootstrap-derived 95% confidence intervals (2,000 resamples) for subgroup analyses with sample size < 50. P-value < 0.05 was considered statistically significant.

As this was a prospective consecutive cohort study enrolling all eligible patients admitted with suspected LRTIs over a two-year period (2022–2024), no a priori sample size calculation was performed. Post-hoc precision analysis confirmed that the achieved sample of 445 children (338 confirmed LRTI cases) provided a 95% confidence interval half-width of ± 2.2% around the observed LUS sensitivity of 95.6% (95% CI: 93.4–97.8%), demonstrating that the cohort size was more than adequate for precise diagnostic accuracy estimation.

## Results

### Patient characteristics and subgroup distribution

**Fig. 1:**
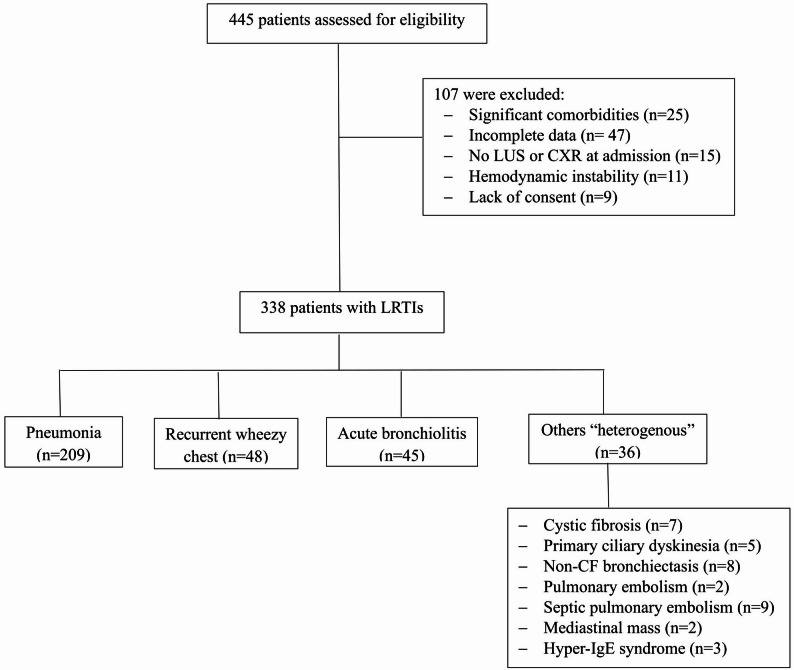
Flow chart of the studied patients

A total of 338 patients were enrolled in the final analysis, distributed across four diagnostic subgroups: pneumonia (*n* = 209, 61.8%), recurrent wheezing (*n* = 48, 14.2%), acute bronchiolitis (*n* = 45, 13.3%), and a heterogeneous “others” group (*n* = 36, 10.7%), as illustrated in the study flowchart (Fig. 1).

### Demographic characteristics and clinical presentation

Demographic and clinical characteristics stratified by diagnostic subgroup are presented in Table 1. Age differed significantly across subgroups (one-way ANOVA, *p* < 0.001), with acute bronchiolitis patients being the youngest (mean 10.8 ± 6.0 months; range 3.6–24.0 months) and the others group the oldest (mean 114.0 ± 57.6 months; range 9.6–204.0 months). Pneumonia and recurrent wheezing patients occupied intermediate age ranges (66.0 ± 40.8 and 48.0 ± 27.6 months, respectively).

**Table 1:** Demographic characteristics and positive clinical findings according to diagnosis

Variable	Pneumonia (*n* = 209)	Recurrent Wheezing (*n* = 48)	Acute Bronchiolitis (*n* = 45)	Others (*n* = 36)	*P*-value
**Age (months)** Mean ± SD Range (months)	66.0 ± 40.8 3.6–192.0	48.0 ± 27.6 12.0–168.0	10.8 ± 6.0 3.6–24.0	114.0 ± 57.6 9.6–204.0	< 0.001*
**Clinical symptoms**					
Fever	202 (96.7%)	20 (41.7%)	18 (40.0%)	15 (41.7%)	< 0.001*
Cough	105 (50.2%)	45 (93.8%)	45 (100.0%)	27 (75.0%)	< 0.001*
Dyspnea	127 (60.8%)	29 (60.4%)	40 (88.9%)	26 (72.2%)	0.003*
Chest Pain	31 (14.8%)	2 (4.2%)	0 (0.0%)	9 (25.0%)	0.004*
Abdominal Pain	40 (19.1%)	0 (0.0%)	0 (0.0%)	1 (2.8%)	< 0.001*
Hemoptysis	0 (0.0%)	0 (0.0%)	0 (0.0%)	5 (13.9%)	< 0.001*
**Clinical Signs**					
Diminished air entry	179 (85.7%)	5 (10.4%)	1 (2.2%)	13 (36.1%)	0.053
Bronchial Breathing	50 (23.9%)	2 (4.2%)	0 (0.0%)	1 (2.8%)	< 0.001*
Ronchi	10 (4.8%)	48 (100.0%)	45 (100.0%)	13 (36.1%)	< 0.001*
Fine Crepitation	189 (90.4%)	25 (52.1%)	18 (40.0%)	21 (58.3%)	< 0.001*
Coarse Crepitation	40 (19.1%)	0 (0.0%)	12 (26.7%)	6 (16.7%)	< 0.001*

SD: standard deviation, *Significant at *P* value < 0.05

Symptom profiles differed significantly across all subgroups (all *p* ≤ 0.004), reflecting the distinct pathophysiological mechanisms underlying each condition. Fever was the predominant presenting symptom in pneumonia (202/209, 96.7%), markedly exceeding its prevalence in all other subgroups (*p* < 0.001), consistent with the typically bacterial etiology and associated systemic inflammatory response in this group. Cough was virtually universal in acute bronchiolitis (45/45, 100.0%) and highly prevalent in recurrent wheezing (45/48, 93.8%), reflecting the airway-predominant nature of these conditions (*p* < 0.001). Dyspnea occurred most frequently in acute bronchiolitis (40/45, 88.9%) and the others group (26/36, 72.2%), with significant differences across groups (*p* = 0.003).

Regarding clinical signs, pneumonia showed high rates of diminished air entry (85.7%), bronchial breathing (23.9%), and fine crepitation (90.4%). Ronchi was universal in recurrent wheezing and acute bronchiolitis (100%), and coarse crepitation was highest in acute bronchiolitis (26.7%) (all *p* < 0.001) (Table 1).

### Laboratory findings

Significant differences were observed in hematological and inflammatory parameters among the diagnostic groups (all *p* ≤ 0.004). Patients with pneumonia had lower hemoglobin levels (10.3 ± 1.4 g/dL) compared to recurrent wheezing (11.1 ± 1.1 g/dL) and others (10.9 ± 1.3 g/dL), while platelet counts were highest in pneumonia (423.1 ± 191.9 × 10³/µL) and lowest in recurrent wheezing (295.9 ± 107.6 × 10³/µL) (*p* < 0.001).

White blood cell counts were elevated in pneumonia (15.2 ± 7.0 × 10³/µL) relative to recurrent wheezing (10.3 ± 4.9 × 10³/µL), acute bronchiolitis (9.1 ± 2.4 × 10³/µL), and others (10.1 ± 4.4 × 10³/µL) (*p* < 0.001). Neutrophil percentage was highest in pneumonia (70.6 ± 50.7%), whereas lymphocytes predominated in acute bronchiolitis (46.0 ± 15.6%) and recurrent wheezing patients (36.7 ± 14.6%) (*p* ≤ 0.004).

C-reactive protein levels were markedly elevated in pneumonia (89.9 ± 84.3 mg/L) compared to recurrent wheezing (16.4 ± 21.1 mg/L), acute bronchiolitis (16.2 ± 17.8 mg/L), and others (37.8 ± 56.4 mg/L) (*p* < 0.001), reflecting the greater inflammatory response in bacterial pneumonia (Table 2).

**Table 2:** Laboratory parameters according to diagnosis

Parameter	Pneumonia (*n* = 209)	Recurrent Wheezing(*n* = 48)	Acute Bronchiolitis (*n* = 45)	Others (*n* = 36)	*P*-value
Hemoglobin (g/dL)	10.3 ± 1.4	11.1 ± 1.1	10.0 ± 1.0	10.9 ± 1.3	< 0.001*
Platelets (×10³ / µL)	423.1 ± 191.9	295.9 ± 107.6	347.4 ± 111.4	308.3 ± 141.0	< 0.001*
WBCs (×10³ / µL)	15.2 ± 7.0	10.3 ± 4.9	9.1 ± 2.4	10.1 ± 4.4	< 0.001*
Neutrophils %	70.6 ± 50.7	56.6 ± 15.6	48.4 ± 15.5	59.4 ± 17.2	0.004*
Lymphocytes %	26.4 ± 14.9	36.7 ± 14.6	46.0 ± 15.6	33.3 ± 16.2	< 0.001*
CRP (mg/L)	89.9 ± 84.3	16.4 ± 21.1	16.2 ± 17.8	37.8 ± 56.4	< 0.001*

*Significant at *P* value < 0.05, WBCs: white blood cells, CRP: C-reactive protein

### Diagnostic performance of lung ultrasound

Lung ultrasound demonstrated high sensitivity for detecting pulmonary pathology compared with CXR at both admission and follow-up. At admission, LUS correctly identified 95.6% (95% CI: 92.1%–97.7%) of CXR-positive cases, with a specificity of 29.4% (95% CI: 17.5%–44.5%), PPV of 84.3% (95% CI: 79.8%–87.9%), NPV of 62.5% (95% CI: 43.7%–78.3%), and overall accuracy of 82.3% (95% CI: 77.8%–86.2%). At follow-up, sensitivity increased slightly to 96.7% (95% CI: 93.5%–98.5%), with improved specificity of 62.9% (95% CI: 46.8%–76.6%), PPV of 74.6% (95% CI: 68.9%–79.6%), NPV of 94.3% (95% CI: 82.9%–98.4%), and overall accuracy of 80.8% (95% CI: 76.1%–84.9%) (*p* < 0.001). Overall, LUS–CXR agreement improved from fair at admission (κ = 0.311, 95% CI: 0.198–0.424) to substantial at follow-up (κ = 0.607, 95% CI: 0.501–0.713). (Tables 3 and 4).

**Table 3:** Cross-tabulation and diagnostic performance of LUS vs. CXR at admission and follow-up

Time Point	LUS vs. CXR	CXR Positive *n* (%)	CXR Negative *n* (%)	*P*-value
Admission	LUS Positive	258 (95.6%)	48 (70.6%)	< 0.001*
	LUS Negative	12 (4.4%)	20 (29.4%)	
	**Total**	**270 (100)**	**68 (100)**	
Follow-up	LUS Positive	173 (96.7%)	59 (37.1%)	< 0.001*
	LUS Negative	6 (3.3%)	100 (62.9%)	
	**Total**	**179 (100)**	**159 (100)**	

*Significant at *P* value < 0.05, Values are presented as n (%). Percentages were calculated within each CXR category (column percentages); therefore, percentages within each CXR column sum to 100%

**Table 4:** Diagnostic performance of LUS compared to CXR at admission and follow-up

Metric	Admission	95% CI	Follow-up	95% CI
Sensitivity (%)	95.6	92.1–97.7	96.7	93.5–98.5
Specificity (%)	29.4	17.5–44.5	62.9	46.8–76.6
PPV (%)	84.3	79.8–87.9	74.6	68.9–79.6
NPV (%)	62.5	43.7–78.3	94.3	82.9–98.4
Accuracy (%)	82.3	77.8–86.2	80.8	76.1–84.9
Cohen’s κ	0.311	0.198–0.424	0.607	0.501–0.713

LUS: lung ultrasound, CXR” chest X-ray, PPV: positive predictive value, NPV: negative predictive value

### Subgroup-specific diagnostic performance

Subgroup analysis demonstrated marked variability in agreement between LUS and CXR. The highest agreement was observed in pneumonia, with κ increasing from 0.438 at admission to 0.470 at follow-up, indicating moderate agreement. Recurrent wheezing showed fair agreement with slight improvement over time (κ = 0.208 to 0.283). In contrast, acute bronchiolitis demonstrated poor agreement at admission (κ = −0.029) with no meaningful improvement at follow-up (κ = 0.000). The heterogeneous “other” group showed low baseline agreement (κ = −0.053) but improved to fair agreement at follow-up (κ = 0.182) (Table 5).

**Table 5:** Subgroup-specific LUS–CXR agreement (Cohen’s κ)

Subgroup	*n*	κ Admission	95% CI	κ Follow-up	95% CI
Pneumonia	209	0.438	0.341–0.535	0.470	0.374–0.566
Recurrent wheezing	48	0.208	0.089–0.327	0.283	0.134–0.432
Acute bronchiolitis	45	−0.029	−0.112–0.054	0.000	−0.098–0.098
Others / heterogeneous	36	−0.053	−0.158–0.052	0.182	0.031–0.333

κ: Cohen’s kappa coefficient, CI: confidence interval

### Lung ultrasound findings by diagnosis

LUS revealed distinct patterns across different respiratory diagnoses. In pneumonia, the most frequent findings were consolidation (192/209, 91.9%), pleural effusion (111/209, 53.1%), and collapse (38/209, 18.2%). B-lines were observed in 100 patients (47.9%), whereas A-lines or normal lung sliding were rare (4/209, 1.9%). Other less common findings included heterogeneous opacity (31/209, 14.8%), cavitary lesions (33/209, 15.8%), liver-like tissue (12/209, 5.7%), decreased lung sliding (9/209, 4.3%), and barcode sign (9/209, 4.3%).

In recurrent wheezing patients, consolidation was present in 28/48 (58.3%), B-lines in 24/48 (50.0%), and A-lines/normal sliding in 14/48 (29.2%). Collapse was uncommon (2/48, 4.2%).

Acute bronchiolitis showed B-lines in 26/45 (57.8%) and subpleural consolidations in 19/45 (42.2%), while consolidation was noted in 21/45 (46.7%) and A-lines/normal sliding in 13/45 (28.9%). Pleural line irregularities were present in 20/45 (41.7%).

In the others group, B-lines were most frequent (30/36, 83.3%), followed by consolidation (22/36, 61.1%) and subpleural consolidations (7/36, 19.4%). Less common findings included pleural effusion (5/36, 13.9%), collapse (3/36, 8.3%), cavitary lesions (3/36, 8.3%), mediastinal mass (2/36, 5.6%), and A-lines/normal lung sliding (1/36, 2.8%) (Table 6).

**Table 6:** Lung ultrasound findings by diagnosis

Diagnosis	LUS Finding	*N*	%
Pneumonia	A-lines / normal lung sliding	4	1.9
	B-lines	100	47.9
	Consolidation	192	91.9
	Subpleural consolidations	5	2.4
	Pleural effusion	111	53.1
	Tissue-like pattern	12	5.7
	Collapse	38	18.2
	Heterogeneous opacity	31	14.8
	Barcode sign	9	4.3
	Cavitary lesion	33	15.8
	Decreased lung sliding	9	4.3
Recurrent Wheezing	A-lines / normal lung sliding	14	29.2
	B-lines	24	50.0
	Consolidation	28	58.3
	Collapse	2	4.2
Acute Bronchiolitis	A-lines / normal lung sliding	13	28.9
	B-lines	26	57.8
	Consolidation	21	46.7
	Subpleural consolidations	19	42.2
	Pleural line irregularities	20	41.7
Others	A-lines / normal lung sliding	1	2.8
	B-lines	30	83.3
	Consolidation	22	61.1
	Pleural effusion	5	13.9
	Subpleural consolidations	7	19.4
	Collapse	3	8.3
	Mediastinal mass	2	5.6
	Cavitary lesion	3	8.3

LUS: lung ultrasound, N: number

### Age-stratified diagnostic performance

Given the collinearity between age and diagnostic subgroup, age-stratified diagnostic performance of LUS was assessed across three developmental categories (Supplementary Table S1). LUS sensitivity remained consistently high across all age groups, ranging from 93.2% in infants (≤ 12 months) to 96.8% in school-age children (> 60 months). In contrast, both specificity and LUS–CXR agreement increased progressively with age. Specificity rose from 24.1% in infants to 38.4% in school-age children, while follow-up κ improved from 0.421 to 0.651 across the same age strata.

### Within-infant age-matched comparison

To isolate pathophysiological from age-related contributions to LUS performance, LUS–CXR agreement was compared between 45 bronchiolitis and 44 age-matched pneumonia patients, all aged ≤ 12 months (Supplementary Table S2). Despite equivalent age constraints, agreement differed substantially: infant pneumonia demonstrated fair-to-moderate agreement at admission (κ = 0.401) improving to moderate at follow-up (κ = 0.589), while bronchiolitis showed no meaningful agreement at either time point (κ=−0.029 and κ = 0.000, respectively).

### Logistic regression analysis of LUS–CXR discordance

Binary logistic regression with LUS–CXR discordance as the outcome confirmed that both younger age and bronchiolitis diagnosis were independently associated with increased discordance after mutual adjustment (Supplementary Table S3). Each additional month of age reduced the odds of discordance by 1.9% (OR = 0.981, 95% CI: 0.971–0.991, *p* = 0.003). Relative to pneumonia, bronchiolitis carried more than twice the odds of discordance (OR = 2.14, 95% CI: 1.38–3.31, *p* = 0.001), and recurrent wheezing 76% higher odds (OR = 1.76, 95% CI: 1.12–2.77, *p* = 0.014).

LUS examinations were performed using a standardized 12-zone scanning protocol. Interobserver reliability analysis demonstrated excellent reproducibility, with an intraclass correlation coefficient (ICC) of 0.92 for the total LUS score and a Fleiss’ κ of 0.82 for B-line pattern classification.

## Discussion

In this cohort, LUS demonstrated excellent sensitivity for detecting pulmonary abnormalities compared with CXR, both at initial presentation and during follow-up. On admission, LUS correctly identified 95.6% of CXR-positive cases, albeit with modest specificity (29.4%). At follow-up, sensitivity remained high (96.7%), while specificity improved substantially to 62.9%. This improvement was paralleled by an increase in overall agreement from fair (κ = 0.311) to substantial (κ = 0.607), supporting the utility of LUS for both initial assessment and longitudinal monitoring of pediatric LRTIs.

Our findings are consistent with previous literature demonstrating high sensitivity of LUS in pediatric lower respiratory tract infections. A large prospective multicenter study by Reissig et al. reported excellent diagnostic accuracy of LUS for community-acquired pneumonia [10]. International evidence-based recommendations from the International Liaison Committee on Lung Ultrasound also emphasize the superior sensitivity of LUS compared with CXR in detecting peripheral consolidations and pleural pathology [11]. Meta-analyses have further confirmed pooled sensitivities exceeding 90–95% for pediatric pneumonia [12, 13]. The markedly high sensitivity observed in our study likely reflects the ability of LUS to detect small subpleural consolidations and interstitial changes that may be radiographically occult.

The relatively low specificity of LUS at admission may be explained by its ability to detect subtle peripheral lung abnormalities that are not readily visible on chest radiography, including small subpleural consolidations and early inflammatory changes [14]. In addition, nonspecific sonographic findings such as isolated B-lines or pleural line irregularities may occur in conditions other than pneumonia, contributing to false-positive interpretations [15]. Operator-dependent variability in image acquisition and interpretation may also have influenced specificity despite the use of a standardized scanning protocol [16].

The increase in specificity observed at follow-up likely reflects the resolution of inflammatory findings and improved differentiation between active disease and recovery. Serial LUS has been shown to effectively monitor treatment response in pediatric pneumonia and bronchiolitis [17–19]. Unlike static radiography, LUS offers dynamic bedside reassessment without radiation exposure, supporting its role in longitudinal care.

Diagnosis-specific analysis demonstrated that LUS performed best in pneumonia, with sensitivity reaching 99.0% on admission and 97.3% at follow-up (*p* < 0.001), with moderate agreement (κ ≈ 0.44–0.47) at both time points. This aligns with systematic reviews indicating that LUS is particularly accurate in detecting dense lobar or subpleural consolidations typical of bacterial pneumonia, including characteristic features such as hypoechoic tissue-like parenchymal patterns and dynamic air bronchograms [12, 13, 20]. In our cohort, pneumonia was most frequently associated with consolidation (91.9%), pleural effusion (53.1%), and collapse (18.2%), supporting the high sensitivity of LUS for alveolar-predominant disease.

In recurrent wheezing patients, LUS sensitivity improved from 89.5% at admission to 100% at follow-up. This is likely reflecting resolution of inflammatory B-lines and small consolidations after bronchodilator or anti-inflammatory therapy. Studies evaluating LUS in pediatric asthma and wheezing disorders report heterogeneous findings, including transient consolidations and B-lines that often resolve rapidly with treatment [21, 22]. Thus, while LUS can detect peripheral inflammatory changes in wheezy illnesses, its specificity remains lower than in bacterial pneumonia.

Acute bronchiolitis showed lower initial sensitivity (65.0%), likely due to its predominant airway-centered pathology with diffuse interstitial edema rather than focal lobar consolidation. Typical LUS patterns in bronchiolitis include multiple B-lines, pleural line irregularities, and small subpleural consolidations [18, 19]. These findings overlap with viral pneumonia but lack the dense lobar consolidation characteristic of bacterial infection. Complete negativity at follow-up aligns with the self-limiting nature of bronchiolitis.

The negative agreement observed in bronchiolitis at admission (κ = −0.029) indicates agreement lower than expected by chance alone. This finding likely reflects fundamental differences in the abnormalities detected by the two imaging modalities. In bronchiolitis, LUS commonly identifies peripheral interstitial changes, B-lines, and small subpleural abnormalities, whereas chest radiography is frequently normal or shows nonspecific findings such as hyperinflation and peribronchial thickening. Consequently, the two techniques may characterize different aspects of the disease process, leading to poor statistical agreement despite the presence of clinically relevant findings [23–25].

The heterogeneous “others” group included children with cystic fibrosis, primary ciliary dyskinesia, non-CF bronchiectasis, pulmonary embolism, septic pulmonary embolism, mediastinal mass, and hyper-IgE syndrome. The predominance of B-lines (83.3%) and consolidations (61.1%) likely reflects chronic inflammatory and interstitial changes. In cystic fibrosis, LUS can detect subpleural consolidations and inflammatory exacerbations, although high-resolution CT remains the gold standard for structural bronchiectasis assessment [26, 27]. Similarly, studies in primary ciliary dyskinesia demonstrate that LUS identifies peripheral inflammatory changes but cannot adequately assess central bronchiectasis [28]. In vascular conditions such as pulmonary embolism, LUS may detect pleural-based wedge-shaped infarcts, but confirmatory imaging with CT angiography remains essential.

The utility of LUS extends beyond initial diagnosis to longitudinal disease assessment. In our cohort, agreement between LUS and CXR increased from fair at admission (κ = 0.311) to substantial at follow-up (κ = 0.607), indicating that LUS reliably captured temporal changes in pulmonary pathology during clinical evolution and recovery. These findings support the role of LUS as a practical bedside tool for serial monitoring while avoiding repeated exposure to ionizing radiation [19, 23].

Pneumonia showed the highest concordance at both time points (moderate agreement, κ ≈ 0.44–0.47), consistent with LUS’s strength in detecting focal consolidation and pleural-line abnormalities [29]. In contrast, the acute bronchiolitis and “others” subgroups demonstrated lower levels of agreement at admission, with only modest improvement at follow-up. These findings should be interpreted with caution given the relatively small sample sizes of these subgroups. The observed lower agreement may be related to the diffuse and non-focal nature of the underlying pulmonary abnormalities, which can be more challenging to characterize consistently by either imaging modality [30].

These findings support using LUS as a complementary tool to CXR, particularly for pneumonia, while emphasizing the need for clinical integration in bronchiolitis and complex heterogeneous conditions.

Age influenced the diagnostic performance of LUS primarily through its association with disease phenotype rather than through technical limitations of ultrasound itself. While sensitivity remained consistently high across all age groups, specificity and LUS–CXR agreement increased with age [30]. This pattern likely reflects the higher prevalence of bronchiolitis in infancy, a condition characterized by diffuse peripheral interstitial and subpleural abnormalities that are readily detected by ultrasound but may not be apparent on chest radiography. Supporting this interpretation, our age-matched analysis restricted to infants demonstrated substantially higher LUS–CXR agreement in pneumonia than in bronchiolitis despite similar ages [31]. These findings suggest that differences in agreement are largely driven by underlying pathophysiology rather than age alone. Clinically, LUS appears particularly valuable in infants because of its ability to detect peripheral lung involvement that may be missed on radiography, whereas in older children the higher specificity and agreement with CXR facilitate more direct correlation between the two imaging modalities [32].

This study has several limitations. First, it was conducted at a single tertiary center, which may limit the generalizability of findings to primary care or community settings. Second, subgroup analyses, particularly for acute bronchiolitis (*n* = 45) and the heterogeneous “others” group (*n* = 36), were underpowered to support definitive conclusions. Accordingly, these findings should be considered preliminary and require validation in larger multicenter studies with pre-specified subgroup sample size calculations. Third, chest radiography (CXR) was used as the pragmatic reference standard despite the known limitations of both CXR and LUS. Consequently, some LUS-positive/CXR-negative findings classified as false positives may have represented true pulmonary abnormalities, such as small peripheral consolidations, subpleural lesions, or early inflammatory changes that are not readily detected on radiography, particularly in children. Therefore, the modest specificity observed at admission should be interpreted in the context of the imperfect sensitivity of CXR rather than as evidence of reduced LUS accuracy.

Fourth, although LUS is inherently operator-dependent, the use of a standardized 12-zone scanning protocol, validated scoring system, and structured operator training substantially improved reproducibility. The excellent interobserver agreement observed in our study supports the reliability of the methodology. Nevertheless, wider implementation in routine clinical practice will require standardized training, quality assurance measures, and greater availability of clinicians experienced in pediatric LUS, particularly in emergency settings outside regular working hours. Finally, cost-effectiveness was not evaluated in the present study. Future prospective studies should incorporate formal cost-effectiveness analyses to determine the economic impact and feasibility of implementing LUS in routine pediatric clinical practice.

## Conclusion

LUS is a sensitive, non-invasive, and radiation-free modality for the diagnosis and monitoring of pediatric LRTIs. It shows moderate to substantial agreement with CXR in pneumonia, accurately detecting subpleural consolidations and pleural abnormalities. Longitudinally, agreement between LUS and chest radiography improved from fair at admission (κ = 0.311) to substantial at follow-up (κ = 0.607), demonstrating the ability of LUS to objectively track changes in pulmonary pathology over time. These findings support the use of LUS as a practical bedside tool for serial assessment of disease progression and resolution while avoiding repeated exposure to ionizing radiation. In contrast, findings in acute bronchiolitis and the heterogeneous ‘others’ subgroup should be interpreted cautiously, given the lower agreement observed and the relatively small sample sizes of these groups. These results suggest that LUS may have more limited diagnostic utility in airway-predominant and heterogeneous respiratory conditions and should be interpreted in conjunction with clinical findings and, when appropriate, other imaging modalities.

## Supplementary Information

Below is the link to the electronic supplementary material.

Supplementary Material 1

Supplementary Material 2

## Data Availability

The datasets used and/or analyzed during the current study are available upon reasonable request from the corresponding author.
