# Supplementary material for: Lung ultrasound versus chest X-ray in pediatric lower respiratory tract infections at a tertiary center: a prospective observational study
Source: Ital J Pediatr. 2026 Jul 23;52:116. doi: 10.1186/s13052-026-02314-6 (PMC13401307; doi:10.1186/s13052-026-02314-6)
Supplement: Supplementary file 1 — Supplementary Material 1 [file 13052_2026_2314_MOESM1_ESM.pdf]

Supplementary Table S1 Age-Stratified Diagnostic Performance of Lung Ultrasound

**Age groups**	**n**	**LUS Sensitivity (%)**	**LUS Specificity (%)**	**κ (Admission)**	**κ (Follow-up)**
Infants (≤12 months)	89	93.2	24.1	0.198	0.421
Toddlers/Preschool (13–60 months)	147	96.1	31.8	0.334	0.618
School-age (>60 months)	102	96.8	38.4	0.389	0.651

κ: Cohen's kappa coefficient

Supplementary Table S2 Age-Matched (≤12 Months) Comparison of LUS–CXR Agreement by Diagnosis

Condition (Infants ≤12 months only)	κ (Admission)	κ (Follow-up)
Bronchiolitis (n = 45)	−0.029	0.000
Pneumonia (n = 44)	0.401	0.589

κ: Cohen's kappa coefficient

Supplementary Table S3 Logistic Regression Analysis of Factors Associated with LUS–CXR Discordance

Predictor	OR	95% CI	p-value
Age (per month increase)	0.981	0.971–0.991	0.003
Bronchiolitis vs. Pneumonia	2.14	1.38–3.31	0.001
Recurrent Wheeze vs. Pneumonia	1.76	1.12–2.77	0.014

Outcome variable: LUS–CXR discordance (disagreement vs. agreement). OR: odds ratio; CI: confidence interval. Pneumonia was used as the reference category for diagnostic subgroup

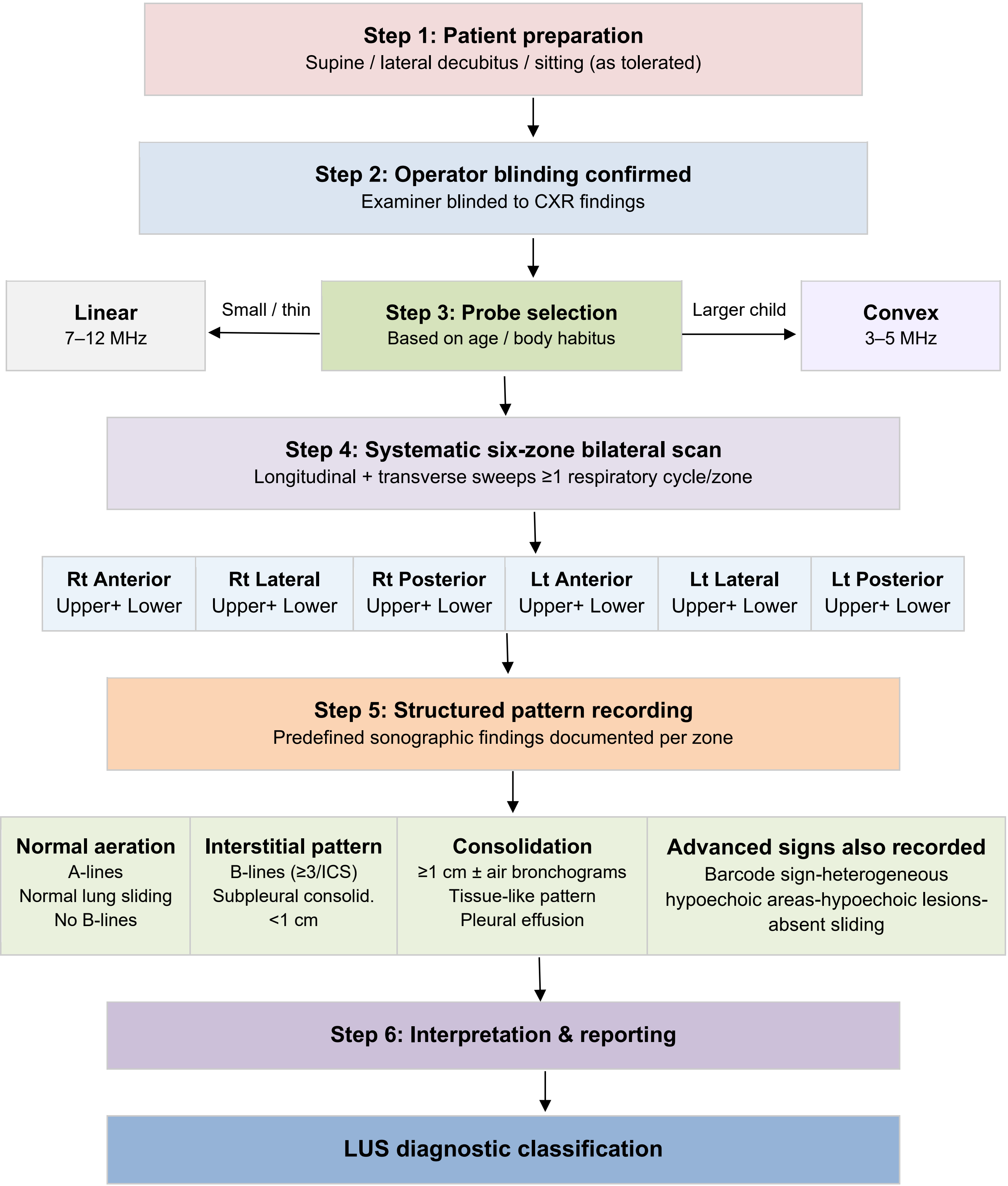

Supplementary Figure S1 Standardized Pediatric LUS Protocol and Diagnostic Workflow
